# 

**Published:** 1898-01

**Authors:** 


*Fifty-Third Year.*

VOL. LIII.
WHOLE NUMBER,
DCXIV.

JANUARY, 1898.

New Series.
VOL. XXXVII.
No. 6.

### BUFFALO MEDICAL JOURNAL

**Established 1845 by AUSTIN FLINT, M. D.**

LIBRARY
SURGEON GENERAL'S OFFICE
JAN.-3--1898

**EDITORS:**

THOS. LOTHROP, M. D. WM. WARREN POTTER, M. D.

**ASSOCIATE EDITORS:**

WILLIAM C. KRAUSS, M. D.

JAMES WRIGHT PUTNAM, M. D.

JOHN A. MILLER, PH. D.

JOHN PARMENTER, M. D.

ERNEST WENDE, M. D.

ALVIN A. HUBBELL, M. D.

SCRIPTA FERUNT ANNOS

**EUROPEAN AGENCY,** J. B. BAILLIÉRE & FILS, 19 Rue Hautefeuille, Paris
*Subscription, if paid in advance, $2.00; otherwise, $2.50. Foreign subscription, $2.50.*
Copyright 1898, by WILLIAM WARREN POTTER, M. D.
Entered at the Post Office at Buffalo, N. Y., as second-class mail matter.

A. T. BROWN PRINTING HOUSE, BUFFALO, N. Y.

**Table of Contents on Page V.**

